# Using Coherence-based spectro-spatial filters for stimulus features prediction from electro-corticographic recordings

**DOI:** 10.1038/s41598-020-63303-1

**Published:** 2020-05-06

**Authors:** Jaime Delgado Saa, Andy Christen, Stephanie Martin, Brian N. Pasley, Robert T. Knight, Anne-Lise Giraud

**Affiliations:** 1 Auditory Language Group, University of Geneva, Geneva, Switzerland; 2 Knight Lab, University of California at Berkeley, Berkeley, USA; 3 BSPAI Lab, Universidad del Norte, Barranquilla, Colombia

**Keywords:** Cognitive neuroscience, Biomedical engineering

## Abstract

The traditional approach in neuroscience relies on encoding models where brain responses are related to different stimuli in order to establish dependencies. In decoding tasks, on the contrary, brain responses are used to predict the stimuli, and traditionally, the signals are assumed stationary within trials, which is rarely the case for natural stimuli. We hypothesize that a decoding model assuming each experimental trial as a realization of a random process more likely reflects the statistical properties of the undergoing process compared to the assumption of stationarity. Here, we propose a Coherence-based spectro-spatial filter that allows for reconstructing stimulus features from brain signal’s features. The proposed method extracts common patterns between features of the brain signals and the stimuli that produced them. These patterns, originating from different recording electrodes are combined, forming a spatial filter that produces a unified prediction of the presented stimulus. This approach takes into account frequency, phase, and spatial distribution of brain features, hence avoiding the need to predefine specific frequency bands of interest or phase relationships between stimulus and brain responses manually. Furthermore, the model does not require the tuning of hyper-parameters, reducing significantly the computational load attached to it. Using three different cognitive tasks (motor movements, speech perception, and speech production), we show that the proposed method consistently improves stimulus feature predictions in terms of correlation (group averages of 0.74 for motor movements, 0.84 for speech perception, and 0.74 for speech production) in comparison with other methods based on regularized multivariate regression, probabilistic graphical models and artificial neural networks. Furthermore, the model parameters revealed those anatomical regions and spectral components that were discriminant in the different cognitive tasks. This novel method does not only provide a useful tool to address fundamental neuroscience questions, but could also be applied to neuroprosthetics.

## Introduction

The traditional approach to investigating brain functions involves the presentation of different stimuli and the analysis of evoked brain response properties^1^. The latter are collected either through non-invasive (e.g., electroencephalography [EEG], magnetoencephalography [MEG] or functional magnetic resonance imaging [fMRI]) or invasive (e.g., electrocorticography [ECoG]) recording techniques^2,3^. Independently of the method used, the measured signals contain background noise arising from other biological processes and environmental interferences that need to be filtered out or attenuated^4,5^. Several preprocessing methods such as signal filtering or referencing can serve to limit neural noise or artifactual activity and are used to improve signals quality prior to the extraction of different brain features. One popular approach in the literature involves the decomposition of brain signals into distinct frequency bands (i.e., delta, theta, alpha, beta, gamma, and high-gamma) broadly divided into low-frequency components (LFC, below 40 Hz) and high-frequency activity. These different frequency bands have been extensively used as features to model brain phenomena^6–17^. For instance, LFCs, usually measured as low-pass filtered brain signal (below 40 Hz)^18–20^, have been used as informative features in several applications, including decoding of position, velocity and acceleration of executed motor tasks^18–26^. The use of this feature produces significant improvements in decoding and prediction compared to models restricted to power modulations in alpha and beta bands^24,25,27^. Given its enhanced signal to noise ratio compared with both EEG and MEG, ECoG can also exploit high-frequency components above 100 Hz. High-frequency band activity (HFB), usually measured as the average power changes in the band from 70 to 200 Hz (although some works include lower spectral components in the range of 60–90 Hz^28^), has been used for decoding in multiple tasks, including motor, auditory, and visual^21,29–34^. The extracted features are used to model brain responses for basic brain research, medical diagnostics, and rehabilitation applications^35–37^. In rehabilitation, brain signal’s features are used to control external devices that allow subjects to interact with the environment^36,38^. In this case, successful use of the devices requires modeling the relationship between the executed task/stimulus and the brain features^3,39^.

Models based on multilinear regression, support vector machines, probabilistic graphical models, and artificial neural networks have been proposed in combination with different types of features^8,9,11–14,18–20,22–26,40–42^. These features involve spatial patterns discovery^28,38,43–47^, which is argued to be critical for increasing signal-to-noise ratio and to improve interpretability of the observed brain activity. Approaches such as common average reference (CAR), spline Laplacian filters and common spatial patterns (CSP) have previously been proposed^48–52^, and more recent approaches based on Riemannian geometry have shown promising results in brain-computer interfaces by using covariance matrices for feature representation and learning^45,53,54^. Modeling of temporal dynamics through probabilistic approaches such as hidden Markov models^42,55–57^, conditional random fields^11–14,21^ and recurrent neural networks^30,31,58^ have also successfully been used. In addition, features based on frequency decomposition of brain signals performed through either Fourier or wavelet analysis are well described in the literature, highlighting the importance of including patient-specific frequency bands in the design of brain-computer interfaces^50,54^. More recently, approaches that go beyond the classic approach based on second order statistics (power spectrum and cross power spectrum) have been introduced, allowing modeling of cross-frequency interactions in brain signals using bi-spectrum^59,60^.

Each method presents advantages and disadvantages. In multilinear regression with distributed lag, a widely used method in the literature^19,23,39,61–68^, the original feature set is expanded by including lagged versions of the original set. Without appropriate regularization, this can introduce model over-fitting^39^. In probabilistic graphical models and deep neural networks architectures, temporal relationships can be incorporated through the modeling of long-range dependencies^21,58^ and include prior information about the execution of the tasks^42^. However, these approaches require the use of a considerable amount of data that is usually not available in experiments with humans, limiting the performance of these methods^69^.

We hypothesize that modeling brain responses as realizations of a random rather than a stationary process should improve the identification of those features that are critical to their generation. We propose a decoding method based on complex coherence that accounts for different parameters such as frequency, phase, and spatial distribution of neural signals. Such an approach does not require manually predefining frequency bands of interest or phase relationships (i.e. lags) between stimulus and brain responses. The method is built on the notion that each experimental trial is a realization of a random process whose characteristics reflect the presented stimulus or executed task. In contrast with methods assuming signal stationary within a trial^19,23,39,61–68^, a statistically consistent phase difference between the neural signals and stimulus is expected across repeated trials of the same stimulus presentation, leaving all other parameters (e.g., specific frequency bands of interest or the phase relationships (i.e., lags values) between stimulus and brain features) hypothesis free. Although other approaches attempted to solve the non-stationarity problem by modeling the temporal dynamics of brain signal’s features^21,42,58^, their performance is limited by the amount of data available for training models. Our results show that the proposed approach significantly improves decoding performance and reduces the computational load compared to other methods that require explicit tuning of hyper-parameters.

The remainder of this document is organized as follows. We first describe the proposed method, experimental tasks, and evaluation criteria, and then present a thorough analysis of the obtained results. We discuss the main aspects of the proposed method based on the results and compare this to other approaches in the literature, including caveats and cautions on the use of the proposed method.

## Methods

### Proposed method

**Figure 1:**
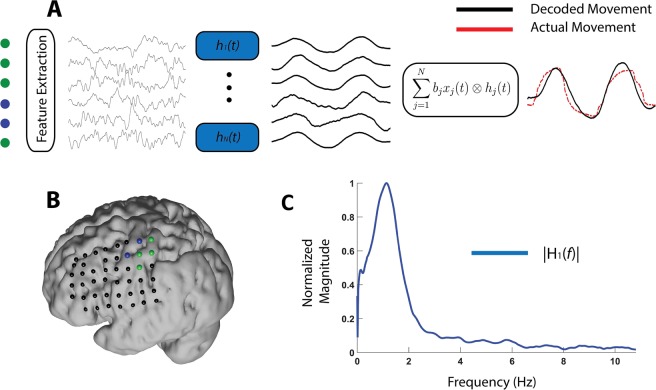
Description of the coherence-based spectro-spatial filter. The diagram represents the recordings of a trial while the patient performs a movement with his/her thumb. (**A**) Brain features are used as input to a set of linear filters trained for each electrode. The filters represent the transfer function of a linear system that maps the brain features to the movement of the finger. The outputs of all filters are combined using linear regression to produce the final prediction of the movement. The signals shown in the figure correspond to LFCs components. (**B**) Example of one patient’s electrodes location. (**C**) Example of the magnitude response of one of the learned filters showing a peak in the low-frequency domain around 1 Hz.

The proposed method is described in Fig. 1 using a finger movement task as an example. To model brain responses to stimulus/task- execution, each trial of an experiment is assumed to be a realization of a random process. Individual trials measure the superposition of the response due to the stimulus/task plus random noise that is assumed to be uncorrelated to the actual brain response of interest. The proposed method makes use of complex coherence to determine the relationship in amplitude and (more importantly) phase between stimuli and brain features at each frequency components across the training trials. In this approach, the coherence is estimated across trials, instead of across time windows in each trial. That is, it is assumed that the properties of the process are the same across trials, but not necessarily across the time course of individual trials. This allows relaxing assumptions about the stationarity of the brain features. For each electrode, a spectral filter is calculated.

The spectral filter extracts commonalities between stimuli and brain features at each frequency band. The output of these filters is then combined using multivariate linear regression, producing a final prediction that incorporates frequency, phase, and spatial features of the brain response. Importantly, spatial filtering results can be analyzed to determine the contribution of different recording electrodes on the prediction, examining for anatomical discriminability among different cognitive tasks or different stimuli.

#### Signal preprocessing and feature extraction

For all three data-sets the preprocessing stage is identical. All electrodes were band-pass filtered between 0.1 and 200 Hz using a zero-phase Butterworth filter of 4^*th*^ order. A notch filter at 60 Hz was used to reduced the interference of the power line (See Supplementary Table S1). A common average reference (CAR) was used to reduce the effect of the reference electrode in all the recording electrodes. For this, the average across all electrodes is subtracted from the individual electrodes in the following fashion:

1 $$z(t)=s(t)-\frac{{\sum }_{k=1}^{N}{s}_{k}(t)}{N}$$

where *s*(*t*) = {*s*_1_(*t*), …, *s*_*N*_(*t*)} represent the brain signals measured for a trial at different electrode locations preprocessed with the bandpass filter (0.1–200 Hz) and the notch filter at 60 Hz. *N* is the number of electrodes.

The brain signals were separated within two frequency ranges: low frequency components (LFC) from 0.5 to 40 Hz, and HFB(70 to 170 Hz). LFCs were estimated by filtering the brain signals with a band-pass Butterworth filter of 4th order between 0.5 and 40 Hz. The envelope of high frequency band components (HFBE) was calculated as the magnitude of the analytic signal in the following fashion: assuming that the signal *z*_*bandpass*_(*t*) is the result of band-pass filtering *z*(*t*) in the HFB range, the analytic signal *z*_*analytic*_(*t*) is calculated as:

2 $${z}_{analytic}(t)={z}_{bandpass}(t)+j {\mathcal H} \{{z}_{bandpass}(t)\}$$

where ${\mathcal H}$ represents the Hilbert transform operation. The envelope of high frequency band components was low-pass filtered with a Butterworth filter of 4^*th*^ order and a cut-off frequency of 40 Hz to reduce rapid changes in the amplitude of the signal. After this, LFCs and HFBE where down-sampled to 200 Hz in order to reduce redundant information. The stimulus features (speech envelope or finger movement dynamics) were accordingly down-sampled to 200 Hz.

#### Coherence-based spectro-spatial filter

The complex coherence allows for determining how well two signals correlate at each frequency component^70^. Given the random variables *x*(*t*) = {*x*_1_(*t*), …, *x*_*N*_(*t*)} representing the brain features extracted at each recording electrode (either LFCs, HFBE or the concatenation of both) and *y*(*t*) representing the dynamics of the stimuli that elicits the brain responses, the complex coherence between each *x*_*j*_(*t*) and *y*(*t*) is given by:

3 $${C}_{{x}_{j},y}(f)=\frac{{P}_{{x}_{j},y}(f)}{\sqrt{{P}_{{x}_{j},{x}_{j}}(f)}\sqrt{{P}_{y,y}(f)}}$$

where ${P}_{{x}_{j},{x}_{j}}(f)$ and *P*_*y*,*y*_(*f*) are the power spectral density of *x*_*j*_(*t*) (brain signal features at electrode *j*) and *y*(*t*) respectively, and ${P}_{{x}_{j},y}(f)$ is the cross-power spectral density calculated between *x*_*j*_(*t*) and *y*(*t*). Power spectral densities (and therefore the complex coherence) were calculated based on the welch method, but rather than dividing each trial in several segments, we assume that each trial of the same class is a different realization of the same process and therefore each trial is one window in the calculation. The windows size is then the length of the trial, the window function used was a Hamming window, and the overlap was set to zero. The magnitude squared of the complex coherence has values between 0 and 1 and can be understood as the squared correlation between the two signals at each frequency component. Using the coherence as a measure of correlation between two signals at each frequency *f*, a linear filter is defined as:

4 $${H}_{j}(f)=\frac{{C}_{{x}_{j},y}(f)\sqrt{{P}_{y,y}(f)}}{\sqrt{{P}_{{x}_{j},{x}_{j}}}(f)}$$

Using the expression in Equation (3) for coherence we obtain:

5 $${H}_{j}(f)=\frac{{P}_{{x}_{j},y}(f)}{{P}_{{x}_{j},{x}_{j}}(f)}$$

where *H*_*j*_(*f*) is the linear filter. Note that ${P}_{{x}_{j},y}(f)$ is a complex quantity, while ${P}_{{x}_{j},{x}_{j}}$ is real. Therefore the phase spectrum of *H*_*j*_(*f*) is given by the phase differences between *y*(*t*) and *x*_*j*_(*t*). That is, for prediction of *y*(*t*) from *x*_*j*_(*t*), the phase differences at each frequency *f* are taken into consideration by the filter. Estimation of *y*(*t*) from *x*_*j*_(*t*) is then obtained by:

6 $$y(t)\cong {x}_{j}(t)\ast {h}_{j}(t)$$

where *h*_*j*_(*t*) represent the inverse Fourier transform of *H*_*j*_(*f*). Finally, different electrodes may contain different type of information, and should be accordingly combined forming a spatial filter, as follows:

7 $$y(t)=\mathop{\sum }\limits_{j=1}^{N}\,{b}_{j}{x}_{j}(t)\ast {h}_{j}(t)+n(t)$$

where *b*_*j*_ is a weight that determines how important the *j*^*th*^ feature *x*_*j*_(*t*) is for the prediction of the signal *y*(*t*). The term *n*(*t*) is used to model the error in the prediction. The set of coefficients *b*_*j*_, can be understood as a spatial filter that provides information about which brain areas are involved in the processing of the stimuli or task executed. Combining *h*(*t*) with the coefficients in Equation (7) forms a filter that takes into account the frequency and phase spectrum of the signals, and the spatial patterns representing the contribution of different brain areas.

It is worth noting that the frequency response of the filters *h*(*t*) is defined by the signals *x*_*j*_(*t*) and *y*(*t*), and therefore should be calculated independently for each patient. Once the filters *h*_*j*_(*t*) are build, parameters *b*_*j*_ can be learned using the least square solution for linear regression. For validation of the performance of the method, the proposed filters are constructed using portion of the available data (training set) and tested in the remaining portion (testing set), using a 5-fold cross-validation approach.

subsectionData-set description.

#### Finger movements

The motor data-set consists of electrocorticographic recordings from five patients that underwent surgery for temporary placement of subdural electrodes due to intractable epilepsy. These data originally appeared in^23^. Clinical information is displayed in Table 1. Patients executed a repetitive finger movement task. During the task, patients were cued with a word displayed on a bedside monitor indicating which finger to move (Thumb, Index, Middle, Ring, and Pinky). Patients were asked to repetitively move the indicated finger during an interval of 2-second (trial). There were thirty trials for each finger. Recordings were done using a Neuroscan Synamps2 device with a sampling rate of 1000 Hz. In addition to ECoG, finger positions were recorded using a 5-degree-of-freedom data-glove sensor. The data-glove signals were originally sampled at 25 Hz and up-sampled at 1000 Hz to match the sampling rate of the ECoG signals. The data-glove signal for each finger is the signal to be predicted from the brain features. Only electrodes in the sensory-motor (S1) and Motor areas (M1) were included in the analysis (See Supplementary Fig. S1). The identification of these areas was based on brain-mapping using electrical stimulation on the patients during the surgery. All patients gave informed consent. The study was approved by the Institutional Review Board of the University of Washington School of Medicine.

**Table 1:** Clinical information for patients in the finger movement data-set.

Patient	Age	Sex	Handedness	Grid Location	Seizure focus
S01	46	F	Left	Left frontal	Left frontal
S02	24	M	Right	Right frontal	Right medial frontal
S03	18	F	Right	Left frontal	Left frontal
S04	32	M	Right	Left fronto-temporal	Left temporal
S05	27	F	Right	Left fronto-temporal	Left temporal

#### Speech perception

The data-set consists of ECoG recordings from three patients that underwent placement of subdural electrodes due to intractable epilepsy. These data originally appeared in^71^. Clinical information is displayed in Table 2. During the experiment, patients were requested to listen to a taped female voice repeating six different words (battlefield, cowboys, python, spoon, swimming and telephone). Each trial started with a baseline period of 500 ms after which a word out of a total of six is randomly selected and played on speakers at the bedside of the patient. Based on the anatomical mapping, electrodes that responded to auditory stimulus were selected (see Supplementary Figure S2). Each word was repeated eighteen times. Recordings were done using a g.USBAMP (g.tec medical engineering GmbH, Austria) with a sampling rate of 9600 Hz -DC coupled-. The audio input was recorded in parallel with brain signals to achieve the minimum loss of synchronization, the selected sampling rate covers the essential portions of the voice spectrum. We use the speech envelope^72,73^ as the feature to be predicted from brain features using the proposed method. For each trial, we selected 1.5 s segments starting from the auditory onset. All patients volunteered and gave their informed consent. The experimental protocol was approved by the Albany Medical College Institutional Review Board and methods were carried out in accordance with the approved guidelines and regulations.

**Table 2:** Clinical information for patients in the speech perception and speech production data-sets.

Patient	Age	Sex	Handedness	Grid Location
P01	NA	NA	Left	Left frontal, temporal
P02	25	F	Right	Right frontal, temporal, parietal
P03	19	F	Left	Left frontal, temporal, parietal

#### Speech production

The data-set consists of ECoG recordings from three patients (same patients as in the perception task with the same recording parameters). During the experiment, patients repeated a particular word presented to them (among six different words: battlefield, cowboys, python, spoon, swimming and telephone). Each trial started with a baseline period of 500 ms after which the patient repeated the word that he or she heard prior to the beginning of the trial. Based on clinical mapping, electrodes that responded speech tasks were selected and used for both speech perception and speech production analysis. Each word was repeated eighteen times. Technical details of the recordings are the same as described in the speech perception data-set. These data originally appeared in^71^. For each trial, we selected 1.5 s segments starting from speech production onset. This onset was determined using an audio signal which recorded along with the ECoG. The experimental protocol was approved by the Albany Medical College Institutional Review Board and methods were carried out in accordance with the approved guidelines and regulations.

### Evaluation

For the finger movement data-set, we compare the results obtained with the proposed method with results available in the literature. 1. Pace-regression: used for finger movement detection in^23^, 2. A probabilistic graphical model presented in^42^ that uses prior information to model the smooth dynamics of the finger movement, 3. A mTRF (multivariate Temporal Response filter) presented in^39^ and, 3. A non-linear function fitting method based on neural networks (named ANNFit in the remaining of the document).

The methods based on Pace-regression and probabilistic graphical models used the same set of data used in this work. Details of the methods and parameter selection can be found in^23^ and^42^. mTRF^39^ learns a multivariate temporal response function that can be used to map brain signal features to stimulus properties or vice-versa, making use of regularized linear regression. In our case, the goal is to predict stimulus properties or task dynamics form the brain signal features. mTRF builds a highly dimensional feature set by adding lagged versions of the original features. The lags can be positive or negative (making the resulting filter non-causal). The brain signal features were lagged using values from −500 to 500 ms, with steeps of 1/*fs* where *fs* is the sampling rate of the features. In order to avoid over-fitting, given the large number of features used, mTRFs uses Ridge regression^74,75^, which uses *L*2 regularization; adding the square magnitude of the coefficients as a penalty to the loss function, favoring solutions with coefficients with small square magnitude. The amount of penalization is controlled by a hyper-parameter (*λ*). We perform a grid search using nested cross-validation within the training set. The range of values for *λ* was selected as presented in a recent study that compares regularization methods in forward and backward models for auditory attention decoding^75^. *λ* values range from 10^−6^ to 10^8^ in 54 logarithmically-spaced steps, using the following formula (Equation 16 in^75^):

8 $${\lambda }_{n}={\lambda }_{0}\times 1.848n,n\in [0,53]$$

where *λ*_0_ = 10^−6^.

A non-linear method based on artificial neural networks was also implemented for comparison (ANNFit). The brain signal feature-set was expanded with lagged versions of the original feature set with lags from −500 ms to 500 ms as for the mTRF method. The first hidden layer is densely connected and uses a sigmoid activation function. In order to avoid over-fitting, a drop-out layer was incorporated with a dropping-out probability of 0.5. The output layer uses a linear combination of the output of the previous layer to predict the finger movement. The artificial neural network was implemented using the Keras library in Python with Tensorflow as backend. We used the mean-squared-error as loss function and 100 epochs. Adadelta was the selected learning algorithm given that it adapts learning rates based on a moving window of gradient updates, making it a robust optimizer compared to other alternatives as Adagrad^76^. The initial learning rate for Adadelta was set to 1.0.

For the listening and speech production data-set, the mTRF method and the ANNFit method were implemented with the same criteria described above for parameter learning and hyper-parameter selection.

The initial set of features input to the models was the same in all cases implemented in this work. While mTRF and the ANNFit models expand the feature set, the proposed method based on coherence does not need to do this, which reduces the probability of over-fitting and reduces the computational load.

We used a 5-fold cross-validation process to evaluate performances. Cross-validation was implemented by dividing the available data for each task in five blocks. In each fold, four blocks were used for training and the remaining block for testing. This process was repeated five times each time having a different block for testing; ensuring that the data in the testing set is not part of the training set within the same fold. For the finger movement dataset a model for each of the five fingers is learned, and a total of 30 trials per finger are available, leaving 24 trials for training and 6 trials for testing on each fold. For the speech datasets a model for each of the 6 words is learned and 18 trials per word are available, therefore in each fold 14 trials were used for training and 4 for testing. In all cases, training or testing, the brain features are accompanied by an external reference signal (data-glove sensor or audio recordings depending of the data-set). These reference signals are used to calculate the filters in the training stage and those in the testing set are employed as targets to validate the output of the proposed method.

Results concerning the prediction of the stimulus features (speech envelope) or task dynamics (finger movement tracking) were obtained using all electrodes previously selected based on anatomical mapping.

## Results

**Figure 2:**
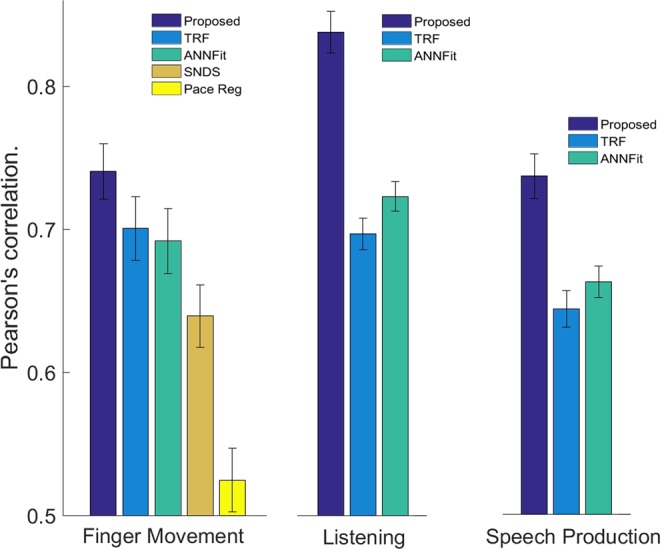
Average performcne in terms of correlation value between the model prediction and the actual stimulus perceived or task executed.

To address whether the coherence-based spectro-spatial filters method outperforms traditional approaches, we first compared its predictive power (in terms of the correlation between the predicted output and the actual stimulus/task dynamics) to the methods described in the Section 2.2. Evaluation of all methods implemented was performed in the same fashion, with the same features, using 5-folds cross-validation. Results show that for the tree data-sets the proposed method based on coherence and spatial filtering provides higher performance as displayed in Fig. 2. To assess the significance of performance gain, we performed a statistical test on the results. For the finger movement data-set, repeated-measures ANOVA on the performance results reveals significant differences between the five compared methods (*DF* = 4, *F* = 27.76, *p* < 0.0001). A post-hoc Tukey-Kramer multi-comparison test shows that the proposed method performs significantly better than all the other methods used for comparison (*p* < 0.001). For speech perception task (Listening) repeated measures ANOVA shows a significant difference between the three methods used (*DF* = 2, *F* = 95.03, *p* < 0.0001). A post-hoc Tukey-Kramer multi-comparison test shows that the proposed method performs significantly better than all the other methods (*p* < 0.0001). Similarly, for the speech production task repeated measures ANOVA shows significant differences in performance between the methods used (*DF* = 2, *F* = 18.53, *p* < 0.0001) and post-hoc Tukey-Kramer multi-comparison test show that the proposed method performs significantly better than the methods used for comparison (*p* < 0.0001). Detailed results for the correlation values between the actual and predicted feature dynamics in all patients across the three modalities are shown in Table 3, Tables 4 and 5 for finger movement task, listening and speech production respectively.

**Table 3:** Averaged correlation values between the predicted and the real finger movement dynamics for the three models.

Patient	Proposed	TRF	ANNFit	SNDS	Pace-Reg	L + H	L + H
	L	H	L + H	L + H	L + H		
S01	0.81	0.67	**0.83**	0.80	0.79	0.65	0.56
S02	0.78	0.68	**0.79**	0.77	0.78	0.67	0.60
S03	0.71	0.48	**0.75**	0.69	0.68	0.65	0.54
S04	0.58	0.39	0.60	0.56	0.54	**0.61**	0.50
S05	0.67	0.62	**0.75**	0.68	0.67	0.62	0.42
Average	0.71	0.57	**0.74**	0.70	0.69	0.64	0.52

L represents low-frequency components (LFC). H represent high-frequency band envelope (HFBE).

**Table 4:** Averaged correlation values between the predicted and the real envelope of speech (speech perception) for the three models.

Patient	Proposed Method			TRF	ANN-fit
	L	H	L+H	L+H	L+H
P01	0.79	0.89	**0.90**	0.71	0.70
P02	0.65	0.75	**0.70**	0.66	0.67
P03	0.80	0.91	**0.92**	0.71	0.79
Average	0.75	0.85	**0.84**	0.69	0.72

L represents low-frequency components (LFC). H represent high-frequency band envelope (HFBE).

**Table 5:** Averaged correlation values between the predicted and the real envelope of speech (speech production) for the three models.L represents low-frequency components (LFC). H represent high-frequency band envelope (HFBE).

Patient	Proposed Method			TRF	ANN-fit
	L	H	L+H	L+H	L+H
P01	0.39	0.67	**0.79**	0.58	0.66
P02	0.70	0.67	0.69	**0.70**	0.62
P03	0.53	0.69	**0.73**	0.65	0.71
Average	0.54	0.68	**0.74**	0.64	0.66

L represents low-frequency components (LFC). H represent high-frequency band envelope (HFBE).

**Figure 3:**
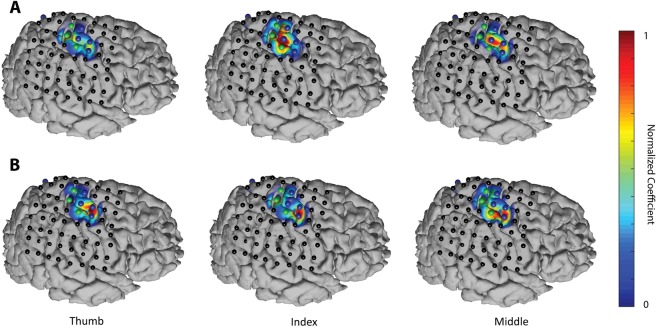
(**A**) Spatial patterns of (**A**) LFC and (**B**) HFBE of a patient (S02) during movement of three different fingers. Filters coefficient have been linearly normalized to range between 0 and 1.

**Figure 4:**
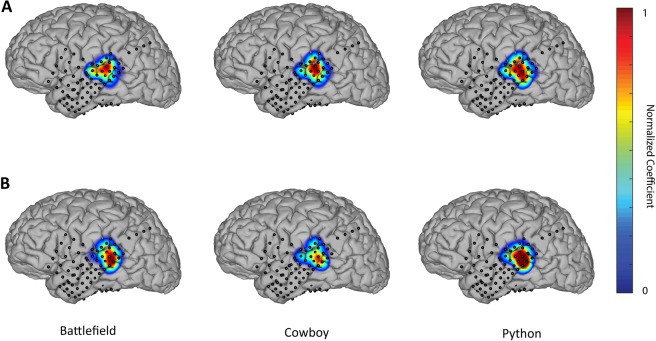
(**A**) Spatial patterns of (**A**) LFC and (**B**) HFBE of a patient (P03) during the perception of three different words. Filters coefficient have been linearly normalized to range between 0 and 1.

**Figure 5:**
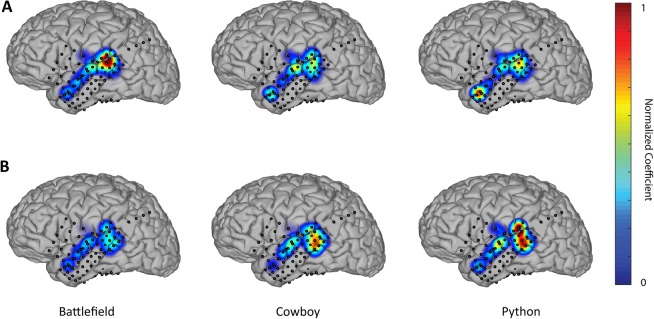
(**A**) Spatial patterns of (**A**) LFC and (**B**) HFBE of a patient (P03) during the production of three different words. Filters coefficient have been linearly normalized to range between 0 and 1.

Furthermore, the proposed method enables to analyze the importance or weight of different recording electrodes in the prediction of the stimulus/task dynamics. As a result, the magnitude of coefficients *b*_*j*_ (see Equation (7)) reflects the contribution of each electrode to the final prediction. In the proposed method, the combination of channels occurs after the filtering stage. Therefore, the output of each filter only contains components that are linearly related to the stimulus/task dynamics. Consequently, the set of coefficients *b*_*j*_ can be understood as a set of spatial filters that contain discriminant information about the areas involved in task execution. These spatial filters can be plotted on the brain models for different tasks. Figure 3 displays the spatial distribution of the coefficients for LFC (panel B) and HFBE (panel A) components in one representative patient of the finger movement data-set. The results show that the proposed method leads to distinct spatial patterns, not only in response to different finger movements but also in the LFC and HFBE components. Similarly, the spatial filter for predicting the envelope of the perceived (Fig. 4) and produced (Fig. 5) speech revealed a level of discriminability across models learned for each word as well as between models learned using LFC and HFBE.

To assess the degree of discriminability within conditions (finger moved or word perceived/produced) revealed by the magnitude of the weights *b*_*j*_, we trained a set of models for each condition using a 5-fold cross-validation approach. We ranked the level of discriminability of the coefficients *b*_*j*_ assigned to each electrode using K-means (k = 5 for finger movement dataset -five fingers- and 6 for speech data-sets -six words-) and fed an LDA (linear discriminant analysis) classifier with only the best two electrodes (selected using only the training set) to prevent over-fitting. The results revealed that the coefficients of the best two electrodes enabled discrimination of the models fitted to each finger movements with a high level of accuracy/Coen’s Kappa for both LFCs (Acc = 83%, kappa = 0.8) and HFB (Acc = 80%, kappa  =  0.76). Similarly, we found that the coefficients discriminated among the models learned for different words in the speech perception (LFCs Acc  =  90%, kappa  =  0.88; HFBE Acc = 94%, kappa  =  0.93) and production (LFCs Acc = 88%, kappa  =  0.86; HFBE Acc = 93%, kappa  =  0.92) tasks. These results show that the method learns for each condition a spatial filter that indicates the importance of a particular electrode in the decoding of the task dynamics. These results demonstrate that the calculated models for each condition are differentiable by the values of the coefficients, evidencing different spatial activations during the execution of different tasks. Using all the selected electrodes for the finger movement data-set we obtain higher classification performance (LFCs Acc = 95%,Kappa = 0.94; HFBE Acc = 92%, Kappa = 0.90), for speech perception task (LFCs Acc = 98%, Kappa = 0.97; HFBE Acc = 98%, Kappa = 0.97) and for speech production task (LFCs Acc = 94%, Kappa = 0.93; HFBE Acc = 99%, Kappa = 0.99). See Supplementary Figs S4–S6 for more details.

**Figure 6:**
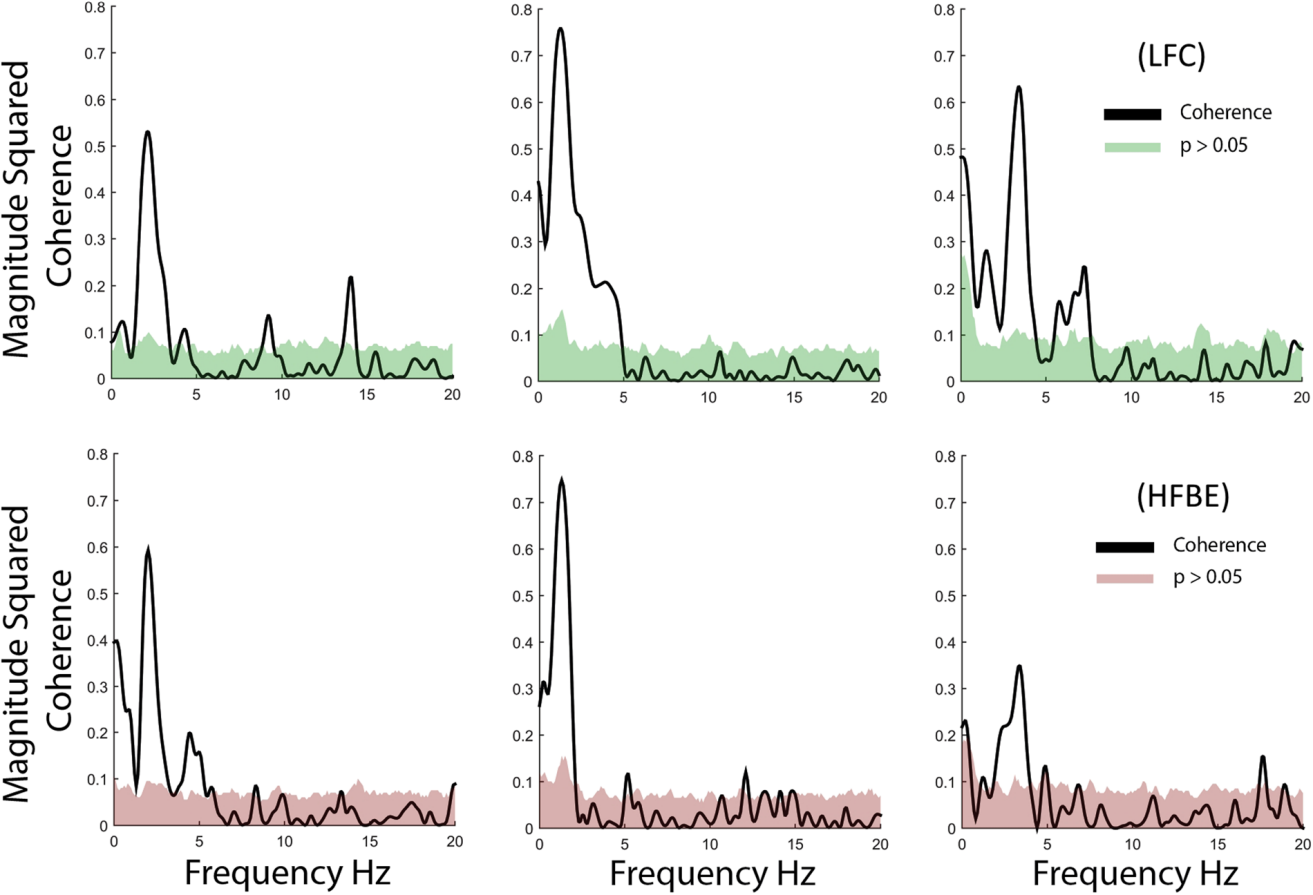
Amplitude squared coherence between the data-glove signals and the brain features in the low frequency (LFC, top panel) and high frequency band envelope (HFBE, bottom). Colored areas show non-significant values for coherence. Note than only portions of the spectrum show significant coherence. The proposed approach appropriately attenuates frequency components that do not contain relevant information.

Figure 6 shows, for the finger movement task, the coherence between the brain features and the data-glove recordings in three patients for the movement of the Thumb. Colored areas indicate statistically non-significant values for coherence at each frequency component calculated using random phase test^77^. It is important to note that the magnitude squared coherence can be understood as the *r*^2^ at each frequency component between the signals into consideration. The peaks with higher value were found to be related to the average rate of finger flexion for individual patients, calculated using the data-glove signals. The High value in the magnitude squared coherence indicates that the brain features are highly informative about the executed task. Importantly, the frequency of peak coherence value is different for each patient. This is an expected observation as patients were cued to flex the finger freely during a particular amount of time. The proposed approach can then calculate the most informative frequency components within the LFC and the HFBE and use those to predict the dynamics of the task while reducing the impact of other components in the final prediction. Furthermore, Fig. 6 also shows a close relationship between the observed coherence when the LFCs and the HFBE are used.

It must be noted that the proposed method does not require the brain features to be in phase across trials. It exploits the fact that the same stimulus will produce a response with the same underlying statistical properties. Therefore, what is exploited, is the consistency in the phase difference between the stimulus and the brain features at each frequency component. This means that even in cases when there is no precise information about the exact moment when the patient starts executing the task, the proposed method will perform well, as is the case for the finger movement data-set and the speech production data-set.

In cases when the stimulus presented is in itself highly non-stationary (as in the speech perception or speech production cases), the traditional approach of using short windows to calculate the coherence is inappropriate, as it assumes that across the time the statistical properties of the signals remain unchanged. In contrast, our approach uses each trial as a realization of the same process, model the relationships at the trial level in the Fourier domain, and then transform the signals back to the time domain. In such cases, physical interpretation of the frequency response of the coherence based filters may be obscured, but the mathematical modeling using Fourier analysis is still valid, and therefore useful for decoding purposes.

Although Person’s correlation is commonly used to report the similarity between the predicted output and the true targets, this index does not take reproducibility into account^78^. To allow future works to be compared with the results presented in this study we included results based on Concordance Correlation Coefficient^78^ (Tables S2–4).

In terms of computational load, the proposed method requires significantly less time to be trained. We calculated the average time necessary to train a model for the movement of the thumb finger with the proposed method and compared it to the mTRF and ANNFIT approaches. The results, after repeating this training 10 times, show a significant difference in the computational load. The proposed method require in average 0.65 +/− 0.07 seconds for training, ANNFIT requires an average of 24.11 +/− 1.4 seconds, and Ridge regression requires 26.1 +/− 1.02 seconds.

## Discussion

### Coherence-based spectro-spatial filter

We propose and assess a method to reconstruct stimuli or task dynamics from features extracted from brain responses, that does not require a priori manual specification of any signal parameters. Relevant parameters are calculated through the use of a reference signal in the training stage and used lately to make predictions based only on the brain features on a patient-specific basis. We found that the coherence-based method outperforms traditional predictive models and provides both a high-performance level (in terms of correlation) and consistency across motor and linguistic domains. The proposed method provides group averages of 0.74 for motor movements, 0.84 for speech perception, and 0.74 for speech production, while the best results obtained among all the other methods used for comparison are 0.7, 0.72 and 0.66 for motor movements, speech perception and speech production respectively. Notably, we demonstrate that the coherence-based method produces spatial filters that are discriminative of the task executed, revealing the importance of different brain areas for the execution of the tasks. The performance improvement relative to other methods is explained by several factors. The proposed method uses frequency decomposition and takes into account the phase relationships across trials. This allows for removing frequency components containing artifactual high power that does not show phase consistency between stimulus and brain features across trials. Such phase relationships are an essential factor because they reflect the latencies between the signals of interest at each frequency component. Multilinear regression-based approaches^19,23,39,61–68^ cannot account for phase relationships and requires expanding the set of regressors with lagged versions of the data which could lead to over-fitting, given the large number of features obtained. mTRF^39^ uses multilinear regression with lags but uses *L*2 regularization to avoid over-fitting issues, which require fine-tuning the regularization parameter *λ* (see 2.2). This makes the method computationally demanding as there is not closed-solution for the value of *λ* and it must be selected using grid search combined with cross-validation or grid search plus model selection approach such as Akaike information criterion (AIC) or Bayesian information criterion (BIC)^79^. This makes mTRF several folds slower than the proposed method for the same task. The proposed filter can be calculated in the Fourier domain using the FFTW algorithm^80^, which is highly efficient and widely implemented in commercial packages, (Python, Matlab, R). The ANNFit method used for comparison includes the possibility to incorporate non-linearities through sigmoid activation functions. However, it cannot model latencies among the signals of interest. Although expanding the regressors set with lagged versions of the data is possible as presented here, and in^23,41,61,62^, this may lead to over-fitting as the number of parameters increases while the amount of available data remains constant. To avoid these issues, we implemented a drop-out layer for the ANNFit method. However, the proposed method based on coherence not only outperforms the ANNFit method but is also computationally more efficient, despite the ANNFit being trained with a backend that uses parallel computing through graphical processing units (GPUs). Recently an approach based on canonical correlation analysis (CCA) has been proposed^41^ with good results. The CCA method produces a transformation of the input signals and also on the output signals (targets), which makes it impossible to compare in the scenario of the data-sets presented here. Nonetheless, the CCA also requires the inclusion of lagged versions of the input or to construct the input using multiple filtered versions of the brain signal features, which makes it prone to over-fitting. (see^41^)

We provided decoding results for LFCs and HFBE used both independently and in combination. For the finger movement data-set we found a high contribution from low frequency components (LFC), as previously reported in the literature^25,81^, which suggests that phase information is the most relevant information to be obtained from LFCs, which is consistent with other works showing selectively phase entrainment of the motor cortex on underlying rhythms in the low frequency range^82^. A recent work^19^ focused on LFCs showed that the phase of the brain features contains more information for decoding of kinematic parameters of the executed movements than the signal amplitude. It is also worth noting that the frequency range of the LFCs varies greatly across the literature, for instance, in^81^ the LFCs have a frequency content up to 20 Hz, while in^24,25^ the frequency content is up to 3 Hz. In^83^ LFCs were set to frequencies below 13 Hz for local field potentials, in^84^ frequencies below 2 Hz were selected for ECoG, and in^85^ a frequency content below 7 Hz was selected for EEG and MEG recordings. The approach in this work was to set a wide band (0–40 Hz) for the LFCs and determines based on coherence the frequencies that show phase consistency with the presented stimulus. The upper band was selected only to avoid contamination due to the power band at 60 Hz. In the case of the two speech data-sets, results show a greater contribution of HFBE compared to LFCs, in the case of speech perception where the brain activity expected is mainly sensory, we expect HFBE to provide better performance as it is highly correlated to the neuronal firing^27^. In the case of speech production there are motor components due to the vocal motor activity and auditory sensory components, therefore it is expected to obtain better performance combining those features. Furthermore, the difference in the performance between speech perception and speech production could be explained by the fact that auditory stimulus are highly consistent across trials as for a particular word, the same audio recording was presented to the patients, while in the speech production, variations in the speed at which the speech is produced were observed in the data. Such variations are difficult to model through linear systems which motivates the use of non-linear approaches as presented in^71^.

Given the limited amount of data available for each patient (30 trials per class for the finger movement data-set and 18 trials per class for the speech data-sets) electrode selection was guided by the clinical mapping, i.e. signal power increase during the relevant (motor or speech) tasks. This basic approach contributes to significantly reduce the dimensionality of the data and to limit over-fitting. While it likely produces interpretable results, it is not necessarily optimal in terms of decoding as areas that are not primarily included might also hold relevant information. When the amount of data is not limited, the spatial component of the proposed filter, can be combined with regularization techniques (i.e., L1 norm) that aim at finding sparse solutions (see^86–88^) allowing automatic selection of relevant electrodes.

The proposed coherence-based filter shows robust performances across different patients and operates well regardless of inter-individual differences and electrodes localization making it well suited for cases where the decoding goal is to decode what stimulus was presented to the patient. Using complex coherence, correlations between stimulus and brain features at each frequency component are calculated and are used to create a filter. Filter parameters are calculated under the assumption that each trial is a realization of a random process which allows employing the different trials to calculate a robust estimation of the cross-spectral density between brain features and the stimulus presented in training data. This permits extraction of components that are in phase with task dynamics across trials for each frequency and recording site. The resulting signal is then combined spatially to form a final prediction. The coherence-based spectro-spatial filter method has the advantage of including different dimensions of brain features such as phase, frequency, and space to handle the prediction of stimulus dynamics in an automated fashion. Importantly, the second stage of the proposed method combines different recordings electrodes. The difference between this and the methods used for comparison is that signals are combined after the coherence-based filtering, which ensures that the components combined have a linear relationship with the stimulus/task dynamics. This reduces the possibility that the coefficients learned (spatial filters) reflect a simple noise-canceling process. Evidence for this is provided by the clustering of the values of the coefficients for each task, enabling discrimination of the models learned by stimulus type (word presented) or task (finger moved), which provides information about the brain areas involved in the particular task.

### Caveats and caution

The proposed method is better suited for discrete tasks. For instance, speech perception experiments as those presented here, in which the patient listens to a word and a prediction of the acoustic envelope of the audio attended is made, is an excellent example of such a discrete task. The proposed method could be used for continuous prediction as long as the causality of the filters *h*_*j*_(*t*) is ensured. Although engineering methods for ensuring causality exists, particular implementations of these techniques are beyond the scope of this study. However, in cases where patients are exposed to long continuous stimuli, methods based on linear switching dynamics or modeling of sequential states like hidden Markov models (HMM), conditional random fields (CRF) and recurrent neural networks, are better options given that the non-stationarity can be modeled with the different states that make part of such models. Although we selected a linear model for spatial filtering, non-linear methods are possible. We selected a linear approach aiming to obtain interpretable results as shown in Figs. 3–5. Finally, as explained before, when the signals that need to be modeled are highly non-stationary, physical or physiological interpretation of the spectral shape of the coherence-based filters should be made with caution as in such cases the Fourier representations of the signals, although numerically correct, may have no meaningful interpretation. Nonetheless, the results are still useful for decoding, as shown in the results of this work, in particular for speech-related tasks.

## Conclusion

We present a method capable of predicting from features extracted from brain signals, characteristic features of a stimulus. The proposed method employs complex coherence to extract common patterns among the brain features related to the dynamics of the presented stimulus. This includes spatial information forming a spectro-spatial filter that is capable of reconstructing the dynamics of the stimulus with high performance (in terms of the correlation coefficient). Analysis of the coefficients that form the learned spatial patterns showed discriminability among different conditions, indicating the involvements of different areas and frequency components during the execution of various cognitive tasks such as finger movement as well as speech perception and production. The anatomical discriminability revealed by the method can be exploited in the design of neuro-prosthesis as well as for investigating the normal brain function.

In order to allow reproducibility of the results, an implementation of the proposed coherence-based method has been made available in^89^.

## Supplementary information

Supplementary Information.

## Data Availability

A Finger movement data-set was made available by Gerwin Schalk. A data-set with the same finger movement task is publicly available at “A library of human electrocorticographic data and analyses.” (https://exhibits.stanford.edu/data/catalog/zk881ps0522). Speech perception and speech production data-sets were provided by Robert T. Knight and Gerwin Schalk and may be provided by them to interested researchers upon request.
