# Supplementary material for: Using Coherence-based spectro-spatial filters for stimulus features prediction from electro-corticographic recordings
Source: Sci Rep. 2020 May 6;10:7637. doi: 10.1038/s41598-020-63303-1 (PMC7203138; doi:10.1038/s41598-020-63303-1)
Supplement: Supplementary file 1 — Supplementary Information. [file 41598_2020_63303_MOESM1_ESM.pdf]

Using Coherence-based spectro-spatial filters for stimulus features prediction from electro-corticographic recordings.

Jaime Delgado Saa[1,3], Andy Christen[1], Stephanie Martin[1], Brian N. Pasley[2], Robert T. Knight[2], Anne-Lise Giraud[1].

Auditory Language Group, University of Geneva[1]
Knight Lab, University of California at Berkeley[2]
BSPAI Lab, Universidad del Norte, Colombia[3]

E-mail: jaime.delgado@unige.ch

Table S1. Averaged correlation values between the predicted and the real finger movement dynamics for the original results and the modified preprocessing, removing the notch filter and using the band of 60-170 for HFBE (LCFs were not modified)

Subject	original results(L+H)	Modified (L+H)
S01	0.83	0.82
S02	0.79	0.80
S03	0.75	0.73
S04	0.60	0.59
S05	0.75	0.74
Average	0.74	0.74

Table S2. Averaged Pearson's correlation values and concordance correlation coefficient (CCC) between the predicted and the real finger movement dynamics.

Subject	Correlation	CCC
S01	0.83	0.78
S02	0.79	0.75
S03	0.75	0.70
S04	0.60	0.53
S05	0.75	0.71

Table S3. Averaged Pearson's correlation values and concordance correlation coefficient (CCC) between the predicted and the real auditory stimulus envelope.

Subject	Correlation	CCC
P01	0.90	0.87
P02	0.70	0.63
P03	0.92	0.88

Table S4. Averaged Pearson's correlation values and concordance correlation coefficient (CCC) between the predicted and the real envelope of the produced speech.

Subject	Correlation	CCC
P01	0.79	0.73
P02	0.70	0.64
P03	0.73	0.63

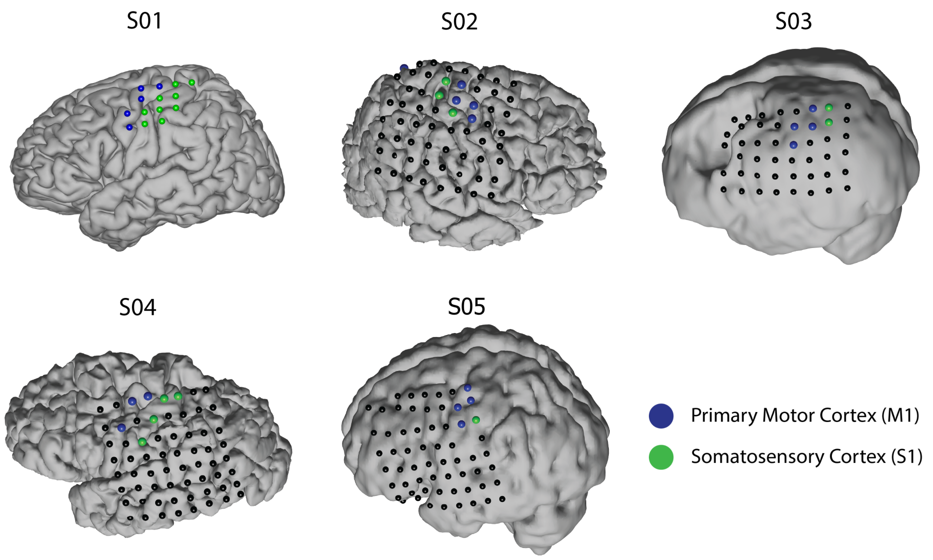

Figure S1. Electrodes locations for all subjects in the finger movement datset. Only electrodes in M1 and S1, -indicated by clinical mapping- were used.

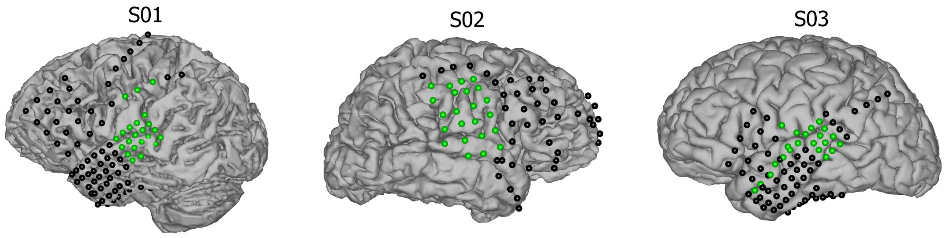

Figure S2. Electrodes locations for speech perception and speech production. Only electrodes that responded to speech during the clinical mapping were used.

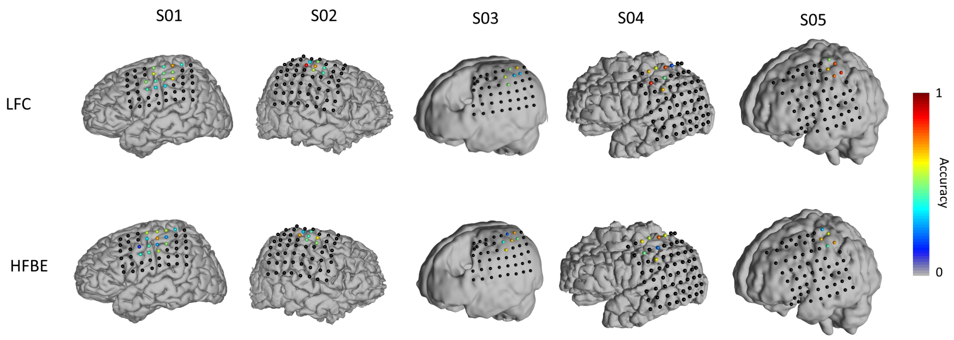

Figure S3. Discriminability among models of each one of the five fingers, per electrode, using LFCs and HFBE. Chance level(20%)

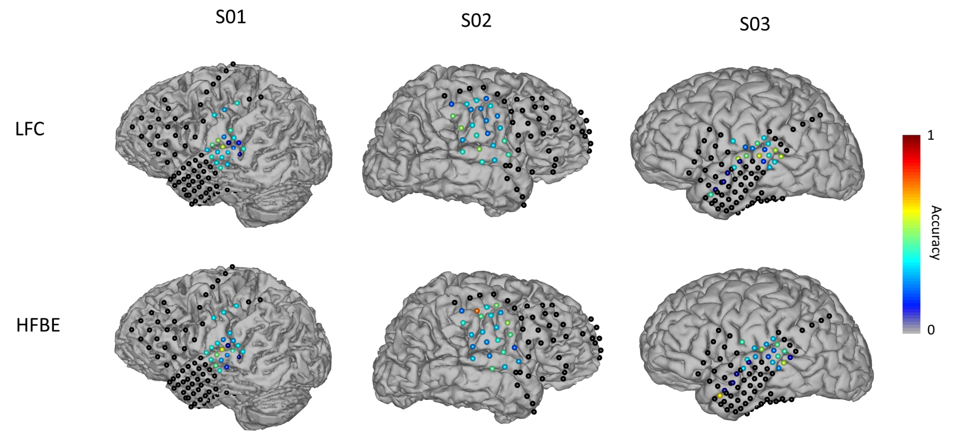

Figure S4. Discriminability among models of each one of the six words, per electrode, for the speech perception data-set using LFCs and HFBE. Chance level(16.7%)

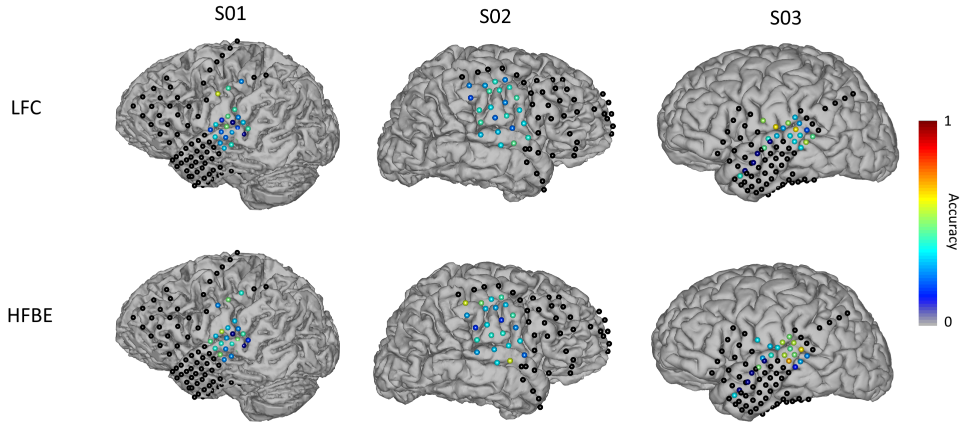

Figure S5. Discriminability among models of each one of the six words, per electrode, for the speech production data-set using LFCs and HFBE. Chance level(16.7%)

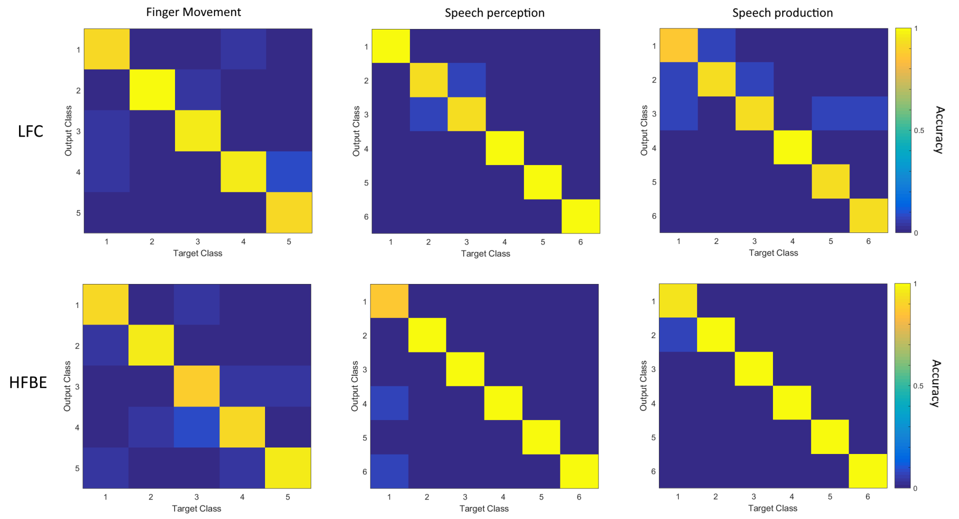

Figure S6. Confusion matrices for model discriminability in the finger movements, speech perception and speech production data-sets.
